# Cutaneous lesions of the external ear

**DOI:** 10.1186/1746-160X-4-2

**Published:** 2008-02-08

**Authors:** Michael Sand, Daniel Sand, Dominik Brors, Peter Altmeyer, Benno Mann, Falk G Bechara

**Affiliations:** 1 Department of General and Visceral Surgery, Augusta Kranken Anstalt, Academic Teaching Hospital of the Ruhr-University Bochum, Germany; 2 Department of Physiological Science, University of California Los Angeles (UCLA), Los Angeles, California, USA; 3 Department of Otorhinolaryngology, Head and Neck Surgery, Ruhr-University Bochum, Germany; 4 Department of Dermatology and Allergology, Ruhr-University Bochum, Germany

## Abstract

Skin diseases on the external aspect of the ear are seen in a variety of medical disciplines. Dermatologists, othorhinolaryngologists, general practitioners, general and plastic surgeons are regularly consulted regarding cutaneous lesions on the ear.

This article will focus on those diseases wherefore surgery or laser therapy is considered as a possible treatment option or which are potentially subject to surgical evaluation.

## Anatomical characteristics

When evaluating skin lesions on the ear, specific anatomical peculiarities should be considered. The outer ear consists of the skin bearing external ear canal and the auricle. Both are of elastic cartilage covered with skin. It is attached to the periost and poorly vascularised. The epidermis on the concave aspect lies on a very thin subcutis which is strongly attached to the auricular cartilage. In contrast the convex aspect of the outer ear has a thicker subcutis with a stronger layer of subcutaneous fat which causes a certain laxity and displaceability compared to the concave side. An additional anatomical uniqueness is the high concentration of holocrine ceruminal glands in the skin of the external ear canal. The cerumen may mask existing diseases of the skin in the entrance of the external ear canal. In case of a ceruminal obstruction, an adequate assessment of the external auditory meatus should be done only after cleaning, which may demask existing dermatosis. The auricle is susceptible to environmental influences and trauma. Because of its exposed localization, the ear is particularly liable to the effects of ultraviolet (UV) light and, consequently, to pre-neoplastic and neoplastic skin lesions. Further, it has a sound-transmitting function and is located at a visible, esthetically obvious site, drawing considerable attention from the patient. Depending on the localization, lesions on the external ear which lead the patient to seek professional help are noticed by the patient himself or by a relative or friend.

When hidden areas of the outer ear are affected, consultation may be delayed until very late in the disease process. This is especially true for malignant tumors which may often present at an invasive stage, due to the minimal thickness of the skin compared to other parts of the body. In many cases, optimal medical care for patients with skin diseases of the external ear requires an interdisciplinary approach dermatological, ear-nose-throat and surgical collaboration. Below, the most important and frequent skin diseases of the ear which are potentially subject to surgical or laser therapy are described. Because of the large number of different diagnosis a description of all pathologic conditions of the external ear seems to be impossible. Hence, we limited our description to the diseases which are frequent or call for special attention because of their prognosis.

## Epithelial tumors of the external ear

### Non-malignant tumors

#### Seborrhoic keratosis (Syn.: seborrhoic wart, senile wart, and basal cell papilloma)

**Figure 1:**
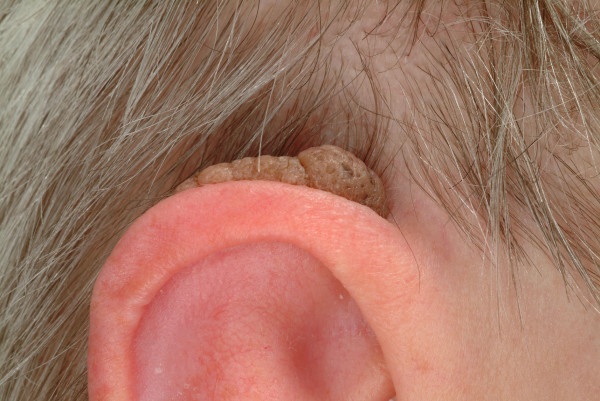
Seborrhoic keratosis. Brownish, exophytic tumor with a velvety to finely verrucous surface. Location on the retroauricular site with partial spreading on the helical rim.

**Seborrhoic keratosis **is one of the most common non-malignant tumor of the external ear. It appears as a light brown, mostly flat, sometimes exophytic papular lesion which originates from proliferative epithelial cells (Fig. 1). Its spread increases with age and can potentially affect the whole ear, including the external auditory canal [1]. Ultraviolet light exposure, human papillomavirus infection, hereditary factors, action of oestrogen and other sex hormones are among those factors which have been suggested in the aetiology of this disease [2]. Secondary malignant changes may occur but are extremely rare [3]. Although treatment varies from pure trichloroacetic acid, cryotherapy to electrodessication, we prefer simple curettage or excisional surgery. Since it may be confused with malignant melanoma or squamous cell carcinoma, obtaining a specimen for histology is essential. Histologically this lesion can be devided into seven subtypes: acanthotic; hyperkeratotic; adenoidal or reticulated; clonal; irritated; inverted follicular keratosis; and melanoacanthoma variants [3]. Especially irritated types of seborrhoic keratosis can be misdiagnosed as squamous cell carcinoma as they frequently show active cellular appearances and a downward proliferation of the active epithelial cells. Therefore a sufficient amount of biopsy should be sent for histologic evaluation in cases of macroscopically suspicious lesions which cannot be clearly evaluated as a common seborrhoic keratosis.

#### Atheroma (Syn.: sebaceous cyst, atheroma, steatoma, keratinous cyst)

**Atheroma **is a benign tumor which is mostly located at the back of the earlobe. On clinical exam, it appears as a 5 – 25 mm firm, displaceable nodule and may show signs of secondary infection. Sometimes, a pinpoint depression at the surface of the cyst corresponds to the infundibulum of a pre-existing hair follicle. The high density of sebaceous glands over the earlobe predisposes the ear for this lesion. Therapy consists of spindle-shaped excision to prevent recurrence. Other techniques of removal include punch biopsy aspiration followed by curettage and avulsion of the cyst wall. Cysts removed from the back of the ears have the highest recurrence rates (13% and 13.8%) [4]. Regardless of the chosen treatment, thorough removal of the cyst wall seems therefore to be essential for reducing the high recurrence rates.

#### Granuloma fissuratum (Syn.: acanthoma fissuratum)

**Figure 2:**
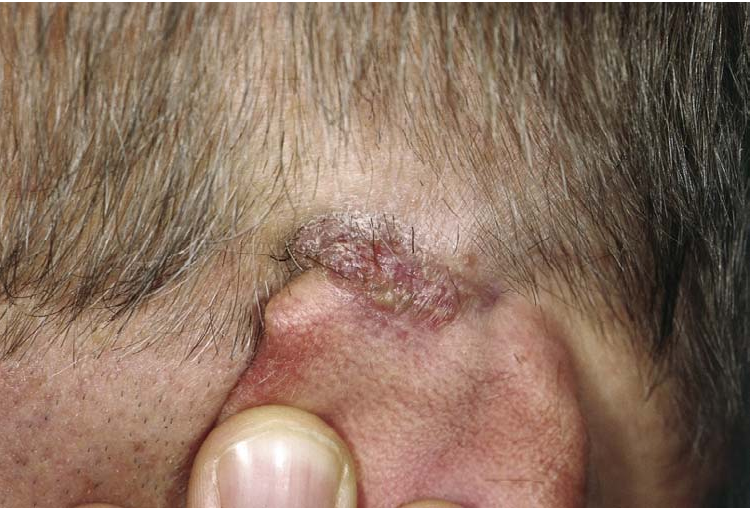
Ganuloma Fissuratum. Skin colored to light red, tender mass of granulation tissue behind the auricle with an exophytic, elliptic growth pattern and a central notch.

**Granuloma fissuratum **is a reactive process of the skin usually caused by chronic trauma from ill-fitting eyeglass frames. The constant pressure of an ill-fitting frame leads nearly always to an unilateral, skin colored to light red, tender mass of granulation tissue behind the auricle with an exophytic, elliptic growth pattern and a central notch (Fig. 2). Its macroscopic appearance has been compared to that of a coffee-bean. It should be kept in mind for resembling malignant tumors. It is a benign differential diagnosis of basal cell carcinoma or squamos-cell carcinoma which can often be managed readily with a correction of the ill-fitting eyeglass frame [5-7]. A few cases are reported in the literature, the exact epidemiologic data is not available as many patients never seek professional help about it.

### Pre-neoplasia

#### Actinic keratoses (Syn.: solar keratoses or senile keratoses)

**Actinic keratoses **is a UV light-induced lesion which is often located on the ear, especially on the helical rim. Its frequency increases with age and can progress to invasive squamous cell carcinoma in 20%, a malignant transformation which treatment can prevent [8]. Its prevalence is higher in individuals with fair complexion. Mostly, a well-defined patch with a rough texture, 3–8 mm in diameter, and typical erythematous base is visible, accompanied by occasional hyperkeratosis. However the lesion may grow to large hyperkeratotic plaques with several centimeters in diameter. Signs of inflammation may occur. In the case of a persistent, recurrent, or isolated lesion, a biopsy is recommended to confirm the diagnosis [9]. Effective treatment options are curettage, photodynamic therapy, laser therapy, topical 5-floururacil (5-FU), diclofenac, colchicine, imiquimod and retinoid application [10-13].

#### Cutaneous Horn (Syn.: cornu cutaneum)

**Cutaneous Horn **is not a pathological diagnosis. A variety of primary underlying processes, benign, premalignant or malignant, can cause this lesion [14-17]. It presents a mostly asymptomatic, variably sized, keratotic mass arising from the superficial layers of the skin or deeply from the cutis [18]. It generally occurs on sites, which are subjected to actinic radiation, with the upper part of the face and the ears being the most common area [19]. In a case series of 643 cutaneous horns, 40% were derived from malignant or premalignant epidermal lesions (squamos cell carcinoma, actinic keratosis), and 60% from benign lesions [17]. The important issue when dealing with this lesion is accurate determination of the nature of the processes at its base. An underlying lesion with malignant or premalignant potential at the base of a cutaneous horn is a common finding wherefore we recommend excision and histology.

### Malignant tumors

#### Basal cell carcinoma (Syn,: basalioma, basal cell epitelioma)

**Figure 3:**
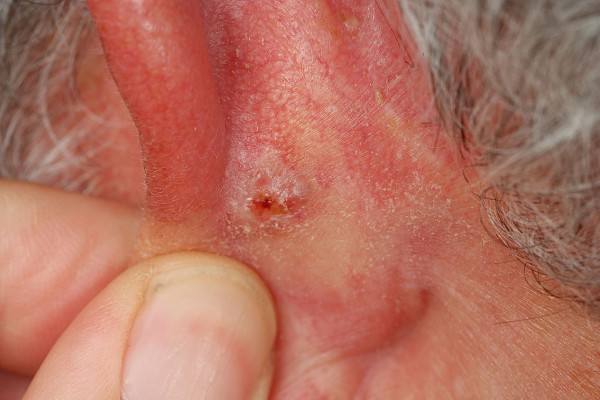
Basalioma. Erythematous papule with indicated pearly border. Remark the central ulceration of the retroauricular located lesion.

**Basal cell carcinoma **(BCC) accounts for 90% of all malignant cutaneous lesions in the head and neck region and is therefore the most common type of skin cancer on the ear. It makes up one fifth of neoplasms that involve the ear and the temporal bone [20]. The vast majority of BCC occurs on the auricular helix and periauricular area which are especially susceptible as they are exposed to the most UV light. Nevertheless 15% arise in the external auditory canal. Five different clinical forms are distinguished in the literature: nodular-ulcerative, pigmented, cystic, superficial multicentric and morphealike. The most common type is the nodular-ulcerative. The lesion is a flesh-colored scaling papule, mostly erythematous to pink, sometimes pigmented, with a surrounding capillary network. It has a pearly border and can show a central ulcer (Fig. 3). This most frequent form may infiltrate the cartilage. Although metastases of BCC are extremely rare, the invasive character of the tumor can cause extensive local tissue destruction. The second most common type is the morphealike or sclerosing subtype. It is more troublesome as it has indistinct margins and infiltrates along deep tissue planes. It spreads centrifugally with a finger-like growth pattern which complicates therapy. The lesion can potentially extend to the temporal bone or parotid gland and remain undetected.

The most successful therapy for basal cell carcinoma is micrographic-controlled surgery (two stage operation). Five-year recurrence rates by micrographic-controlled surgery are reported to be between 1 and 5.6% [21,22]. Nevertheless, BCC found in the middle of the face (so-called *H-zone*), followed by those on the auricular and preauricular area have the highest rate of recurrence following treatment by excisional surgery, radiation, cryosurgery, curettage or electrodessication – all alternative forms of treatment [23-25]. Several theories attempt to explain the high rate of relapse. The ear has a complex anatomy which can confuse the assessment of tumor boundaries [26]. Further an unusual horizontal growth phase makes this tumor prone to incomplete excision [27]. As mentioned above, the skin on the concave aspect of the outer ear is very thin and close to the perichondrium. This encourages subclinical spread [23] as skin cancers grow both radially and vertically. Additionally numerous embryonic fusion planes in the auricular skin have been suggested that may contribute to the spread of the tumor [24]. Pensak has described cartilaginous fissures (Santorini) in the lateral floor of the ear canal and a bony dehiscence (*Huschke's *Foramen) in the medial floor of the ear canal to provide pathways for intracranial tumor spread which also have to be considered [28]. In cases of growth into the parotid gland, a lateral parotidectomy with monitoring of the facial nerve has to be performed. Closure of skin defects can be achieved by local flaps in most patients.

#### Bowens disease (Syn.: Morbus Bowen, carcinoma in situ, squamous intraepidermoid neoplasia)

**Bowens disease **is an intraepidermal *carcinoma in situ*, presenting the preinvasive form of squamous cell carcinoma. It is strongly associated with sun exposure and lesions are in up to 83% infected with human papillomavirus (HPV) type 16 [29]. The lesions are erythematous, scaly patches or plaques with irregular borders which can occur anywhere on the skin. They can become hyperkeratotic, crusted, fissured, or ulcerated and generally occur in sun-exposed areas. On the ear, they are most frequently found on the helical rim or the external side of the auricle. Although the size is variable, Nordin et al. describe a mean size on the ear of 18 mm (range 5–70 mm) [30]. Bowens disease is a *carcinoma in situ *of the epidermis and therefore potentially malignant. Progression to invasive SCC is noted in approximately 10% of Bowen's lesions. It should therefore be completely excised when possible by means of micrographic guided surgery.

Histological the atypical and disordered keratinocytes in bowens disease extend down the follicular epithelium. Superficial, topical treatment is therefore associated with an increased probability of recurrence.

Topical imiquimod, 5-FU, cryotherapy, photodynamic therapy, x-ray and grenz-ray radiation, cauterization or diathermy coagulation therapy are described to be effective but lack mircrographic control [31-33]. The latter forms of treatment can be considered for large lesions which are sometimes spread over the whole ear or for patients who refuse surgery.

#### Squamous cell carcinoma

**Figure 4:**
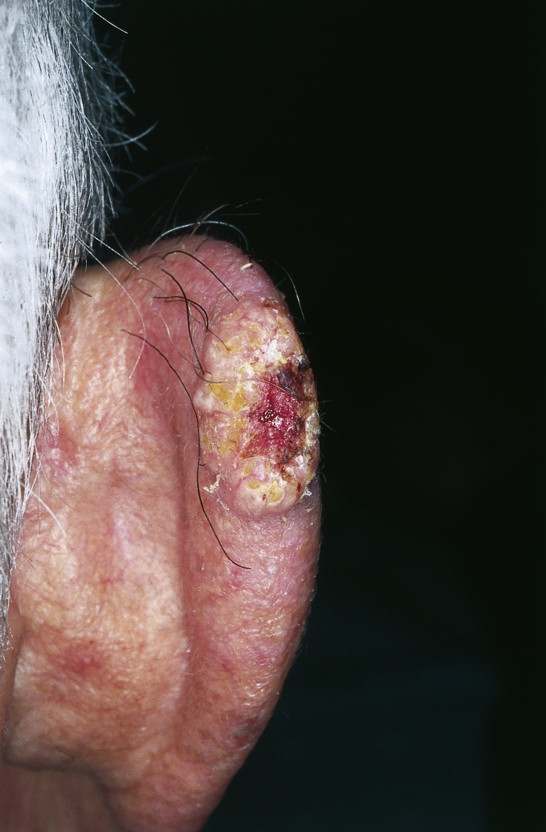
Squamous cell carcinoma. Exophytic, hyperkeratotic tumor with central ulceration, accompanied with seroanguinous exudate.

**Squamous cell carcinoma (SCC) **can arise anywhere on the outer ear and potentially involves the middle ear and the lateral skull base. Nevertheless the tumor mostly originates on the helix and anthelix margin where the skin receives the greatest actinic exposure. Patients are in their 5^th ^to 6^th ^decade of life whereas lesions originating primarily from the external auditory canal generally present 10–15 years earlier. From all patients with SCC of the head and neck, 24% involve the ear and the temporal bone. Sun exposure, fair complexion, cold injury, radiation exposure and chronic infection as well as an association with HPV induced viral carcinogenesis are among the predisposing factors [20,34,35]. The tumor is a scaly, indurated, irregular maculopapular lesion which shows an exo- or endophytic growth pattern with a hyperkeratotic or ulcerating surface, sometimes accompanied by seroanguinous exudates (Fig. 4). When originating from the external auditory canal hemorrhagic otorrhea, falsely treated as otitis externa, is common. Suspicion should arise and biopsy is mandatory whenever otitis externa fails to respond to adequate conservative therapy.

SCC lesions on the nose and ear have the highest rates of recurrence which might be due to an association with embryonic fusion planes [36]. Therapy should therefore be aggressive as tendency of recurrence is high [37]. A complete excision by means of micrographic surgery with tumor free margins is necessary for a successful outcome and should be attempted whenever possible. Although this tumor tends to grow in a vertical fashion it is less likely to respect the barriers of cartilage and bone than BCC. Consequently intratemporal spread with involvement of the external auditory canal is possible and can lead to conductive hearing loss. With further deep extension facial nerve palsy due to destruction of the facial nerve along its vertical or tympanic segment may evolve, and finally a further advancement into the internal auditory canal and cerebellopontine angle may cause dizziness and/or sensorineural hearing loss.

Additionally it is important to investigate for possible regional lymph node metastases which portends poor prognosis. Locoregional metastases follow the lymphatic drainage patterns which include the parotid and upper cervical nodes [38,39]. Nodal involvement is reported to be present in 1–12.5% of all cases [40-42]. Therapy for locoregional metastases is regional lymphadenectomy (Neck-dissection level I-V), followed by postoperative irradiation. It has been suggested that with evidence of lymphovascular or perineural spread in the primary specimen the nearest "sentinel node" should be examined. In cases of histologically aggressive malignancy prophylactic lymphadenectomy and/or regional irradiation should be considered [43,44]. However large multi-institutional studies are missing, therefore the role of sentinel lymph node biopsy for SCC of the head and face region can not be determined so far.

## Non-epithelial tumors of the external ear

### Non-malignant tumors

#### Keloid

**Figure 5:**
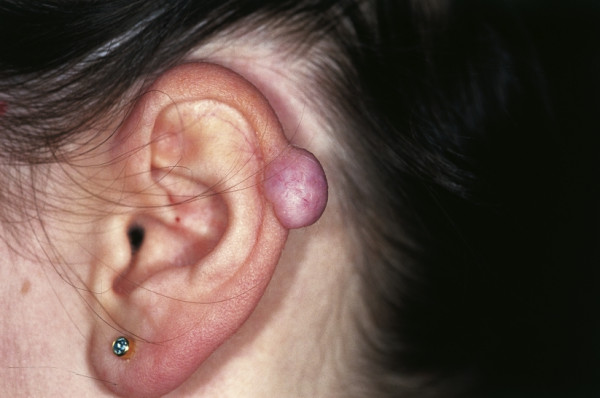
Keloid. Flesh colored to reddish to slight purple nodule on the helical rim. The exophytic tumor shows a smooth surface.

**Keloid **is first described in the Smith Papyrus from ancient Egypt [45]. It is derived from the word *cheloide *which was in the modern languages first mentioned by the French physician Noël Retz in 1790 and later described by Jean Louis Alibert in 1816 [46,47]. It is composed of the Greek words chele (ҳηλη), meaning crab's claw, and the suffix -*oid*, meaning like. Keloids are dermal fibrotic lesions which are considered an aberration of the wound healing process. They are included in the spectrum of fibroproliferative disorders and commonly affect the ears. Clinically dense dermal scar tissue projects above the surrounding skin which is sometimes tender or pruritic (Fig. 5). Keloids on the ear can sometimes be pedunculated. Histology shows thick hyalinized collagen bundles, abundant ground substance, few fibroblasts, and few if any foreign body reactions. They are common after small skin excisions, ear piercing, drainage of auricular hematomas, repair of other auricular traumas, viral infection (smallpox, and herpes varicella-zoster) or as secondary keloid formation after prior keloid excision. In a review of 1200 pierced ears, Simplot et al. report a keloid formation in 2.5% [48]. Several procedures have been described for effective treatment of post-surgical keloid scars. They include silicon occlusive dressings, mechanical compression, radiation, cryosurgery, topical Imiquimod application, bleomycin tattooing, intralesional injections of steroids, 5-floururacil, as well as interferon-alpha, -beta or -gamma in combination with excisional surgery [49-55]. Although optimal conditions for the prevention of keloid formation are still unknown the combination of excisional surgery and the placement of a silicone gel sheet over the wound surface with the application of light pressure are known to be advantageous [56-58].

### Pre-neoplasia

#### Lentigo maligna (Syn.: Hutchinson's freckle)

**Lentigo maligna (LM) **is a slow-growing, non-invasive melanoma in situ. Little attention is paid to this insidious lesion which can potentially become an invasive lentigo maligna melanoma with a conversion rate of 33–50% [59]. The estimated lifetime risk of LM progressing to LM melanoma is 5% [60]. The lesion begins as an unevenly pigmented and irregularly bordered, brown to black macule which slowly extends in the course of time. The lesions size can sometimes obtain several centimetres. It begins as a tan macule which extends peripherally within the course of several years. Non-surgical therapy such as cryosurgery, radiotherapy, electrodessication and curettage, laser surgery, and topical medications with a recurrence rate ranging from 20 to 100% at 5 years have been described in the literature. Recurrence following standard therapies is common because histologic evaluation can be difficult due to the widespread atypical melanocytes that are present in the background of long-standing sun damage [59]. Whenever excision by means of micrographic-controlled or MOHS surgery is possible it should be the preferred method of treatment as it shows the lowest recurrence rate (4–5%) and the best form of margin control among all described forms of therapy. As this lesion occurs more frequently in an elderly patient population, alternative forms of treatment, such as radiotherapy, have to be considered when patients present with very large lesions that are not subject to reconstructive surgery.

#### Malignant tumours

##### Melanoma (Syn.: malignant melanoma)

Approximately twenty percent of all primary **melanomas **are located at the head and neck, of which 7–14% are located at the ear's helix and antihelix. Peripheral parts of the ear are more frequently affected. Interestingly the left ear is more often affected than the right ear. The most accepted theory for this phenomenon is the asymetric UV-dosage in anglo-saxon countries with left-hand driven cars. Further, a male predisposition of 61.5–90.5% is reported in the literature with a predisposition for fair-skinned individuals [61-66]. It can be explained with different hair styles which correlate with UV exposition. With the exception of young children this disease affects all age groups. The average age is 50 years.

**Figure 6:**
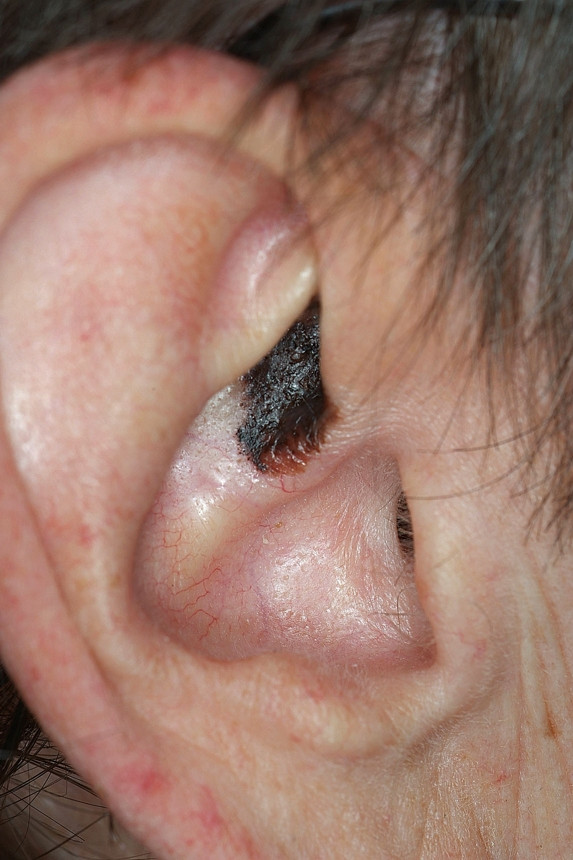
Superficial spreading melanoma. Dark and flat macule with variegated colors. Its borders are irregular, with indentations and notches.

The patients report an asymmetric flat hyperpigmentation or a raised nodular lesion which has changed in color and size. Amelanotic (non-pigmented) variants exist as well. The three most described subtypes are the superficial spreading melanoma, the nodular melanoma and the lentigno maligna melanoma. Each type has its characteristic growth pattern with a horizontal and a vertical growth phase. All over the body, the superficial spreading melanoma is the most common type (Fig. 6). It shows an intermediate radial growth phase before starting to invade the dermis.

**Figure 7:**
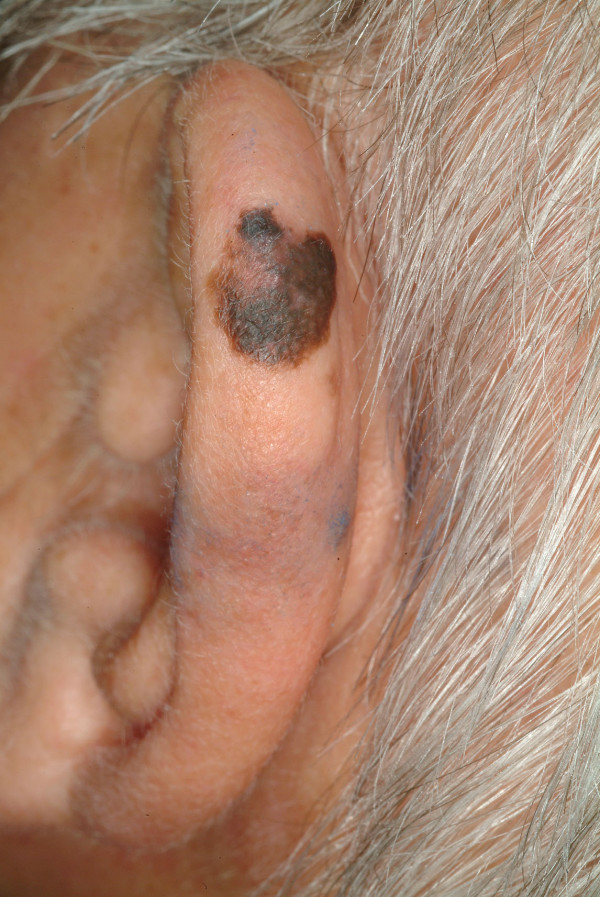
Lentigo maligna melanoma. Irregular pigmented and bordered, brown to black macule with visible bright to reddish regression zone.

A prolonged radial growth phase is characteristic for Lentigo maligna melanoma. A study by Koh et al showed that 8.3% of all head and neck lentigo maligna melanomas occur on the ear [67]. It begins as a macular lesion with a variable pigmentation with an uneven irregular border which shows a sudden increase in size, induration and darkening of the lesion (Fig. 7). The most aggressive melanoma type is the nodular variant, which is a rapidly growing dark pigmented nodule which invades the dermis early in the disease course. The thin layer of subcutaneous tissue contributes to the distinctive invasiveness and therefore bad prognosis for melanoma on the ear. Key pathologic prognostic features of auricular melanomas include the histological subtype, tumor thickness, level of invasion and presence of ulceration. Jahn et al. showed that age, locality, tumour thickness, histological type, level of invasion and excision margins to be significant risk factors for local recurrence [68]. Nevertheless the overall survival of patients with melanoma on the ear depended only on the tumour thickness and Clark level of invasion. Therapy is a surgical approach and in some instances adjuvant therapy. Recommended excision margins are 10–20 mm for primary nodular melanoma (NM) or superficial spreading melanoma (SSM) and 5 mm with complete threedimensional histology of excision margins (3D histology) for lentigo maligna melanoma (LMM). The World Health Association requires a safety margin of 5 mm for melanoma *in situ *and 20 mm for melanoma which are >2.1 mm in vertical thickness. Recent studies have shown that margins > 10 mm have the lowest risk of recurrence [68]. In recent years the more aggressive surgical approach has changed towards narrower excision margins as it has been shown to have only an effect on the incidence of local recurrence and only little impact on disease specific survival.

The available data for sentinel node sampling do not permit definitive conclusions regarding a prognostic or even therapeutic impact of sentinel lymph node biopsy (SLNB) in patients with melanoma of the ear. Patients with tumours thicker than 1 mm are currently undergoing SLNB and should be included in large multicenter studies. In special cases where surgical removal of a lentigo maligna melanoma is not possible, radiation therapy should be considered as an alternative with good results [69]. Unfortunately this tumor is aggressive, with a tendency for spreading to both regional lymph nodes and distant sites. One third of all patients presenting with auricular melanoma have cervical lymph node involvement. As the correlation between melanoma location and drainage is inconsistent lymphoscintigraphy with sentinel node sampling seems to be the primary method of identifying nodal disease [70,71]. However a final evaluation is not possible. Adjuvant therapy includes chemotherapy, immunotherapy, and radiation.

#### Inflammatory lesions

##### Winkler Disease (Syn.: Chondrodermatitis Nodularis Chronica Helicis)

**Figure 8:**
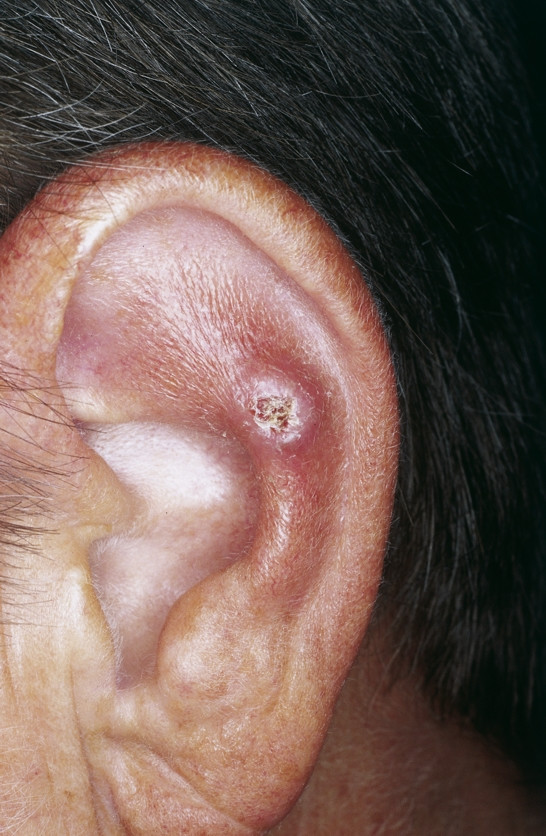
Winkler disease. Ulcerated nodule with overlying crust. The surrounding skin is inflamed as indicated by the red color. Remark: If painful ulcerated nodules are present at the external ear, Winkler disease has to be kept in mind.

**Winkler Disease **is a chronic perichondritis which is thought to be related to limited vascularity at the lateral and anterior aspect of the auricle. The skin is tightly stretched over the underlying cartilage with minimal subcutaneous tissue which results in limited vascularity and ischaemia which is thought to promote the development of this lesion [72]. Mostly located on the helix this disease is characterized by a hard nodule which involves the skin and the cartilage of the ear (Fig. 8). Patients present with severe pain in the affected ear especially when slept on it at night. Although conservative treatment (radiation, topical antibiotics, intralesional steroids) has been described surgical excision should be preferred as lesions show a tendency to recur. A minimal skin excision should be combined with a more extensive cartilage resection to avoid recurrence [73].

##### Lymphocytoma (Syn.: Lymphadenosis cutis benigna)

**Figure 9:**
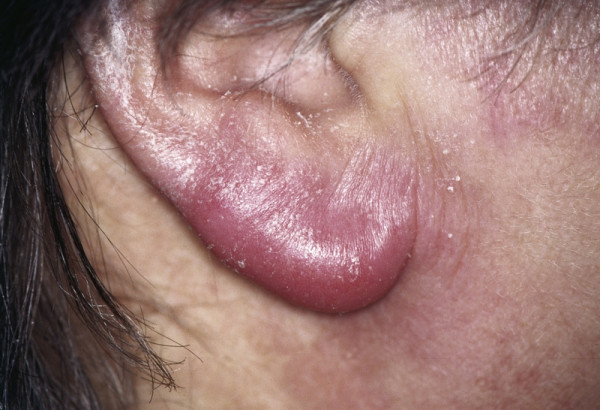
Lymphocytoma. Visible intensely red-violet swelling of the right earlobe.

**Lymphocytoma **can be an early manifestation of an infection with *Borellia burgdorferi *causing Lyme disease. Initially it causes a characteristic rash, erythema chronicum migrans, which is located at the tic bite area. During the second stage an intensely red-violet swelling of the earlobe is characteristic (Fig. 9). An infection with Borellia burgdorferi is the case in one third of all earlobe lymphocytomas wherefore is has to be ruled out serologically when suspected. The majority (two third of all cases) are idiopathic. Antibiotic therapy consists of doxycyclin p.o. for 2–3 weeks. When the lesions do not improve under antibiotic treatment pseudolymphoma is one possible differential diagnosis. Small lesions can be excised.

#### Infectious lesions

Infectious lesions of the external ear are rarely subject to surgical intervention. Nevertheless drainage of the subperichondral space and surgical removal of necrotic ear tissue following infectious diseases of the external ear are sometimes necessary.

**Figure 10:**
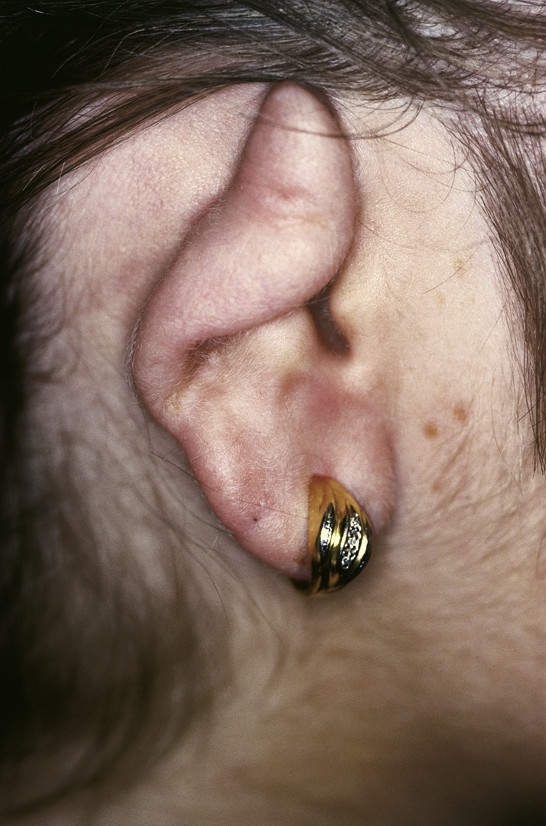
Polychondritis recidivans. Visible deformity of the ears cartilage, resulting from recurrent chondritis.

**Auricular chondritis and perichondritis **is an infection of the auricle. Clinically the ear appears erythematous, tender and a fluctuant swelling is mostly present. Typically an inciting event (piercing, surgery, trauma, wrestling, acupuncture) is followed by an infection resulting from the collection of blood or serum in the subperichondrial space [74]. The most common organisms which have been tested as causative are *S. aureus*, *P. aeruginosa *and *Proteus *species [75]. The subperichondral space must be surgically evacuated and antibiotic therapy consisting of an antipseudomonal aminopenicillin or a flourquinolone for a period of 2–4 weeks, applied [76]. As a result of recurring chondritis persisting deformities of the ears' cartilage can remain (Fig. 10).

**Figure 11:**
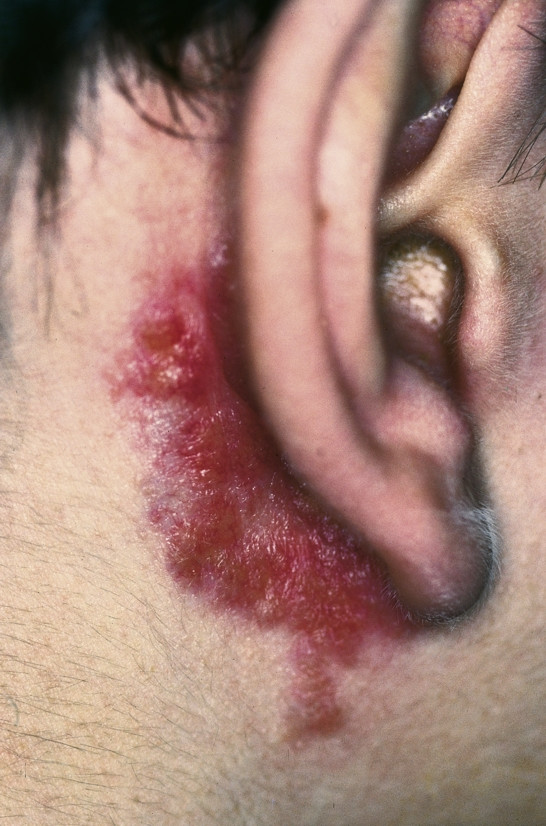
Cutaneous tuberculosis. Retroauricular located red, partially brownish plaque with smooth surface.

**Lupus vulgaris **is a persistent form of cutaneous tuberculosis which potentially involves the ear. Lesions are sharply defined and brown, with a gelatinous consistency on erythematous base (Fig. 11). Therapy is a combination of antibiotics (isoniazid, rifampicin, pyrazinamide and ethambutol) given over a period of several months, and surgical excision of necrotic tissue. As tuberculosis is enjoying a renaissance in western countries the incidence of cutaneous tuberculosis will increase in the future.

#### Rare Lesions

##### Cylindroma (Syn.: Cylindroma, Spiegler's Tumor, Turban Tumor)

**Figure 12:**
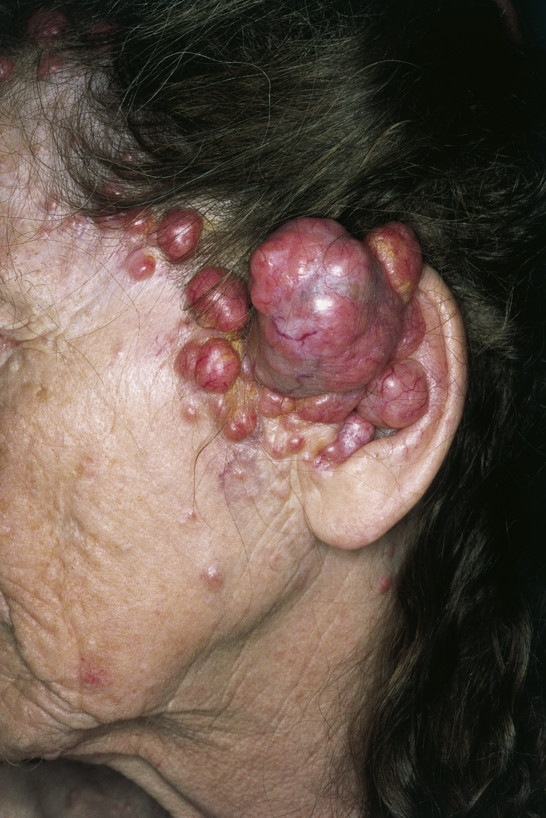
Cylindroma. Numerous pink, red, and partially bluish, firm nodules, affecting the upper parts of the ear and spreading to the left cheek. The distribution and arrangement of tumor masses resemble a bunch of grapes.

**Cylindroma **is benign, solitary or group-like skin colored or light red, bulging, protuberand tumors with a flat, shining surface. They are usually located at the head and neck (turban tumor) and can potentially involve the ear (Fig. 12). They most likely represent very primitive sweat gland tumors originating from eccrine or apocrine glands. Histologically they show apocrine, eccrine, secretory, and ductal features, and the exact cellular origin of cylindromas remains unknown. Although benign, malignant transformation (cylindrocarcinoma) has been reported, in which case surgical excision is the treatment of choice [77].

##### Adnexal Tumours (Syn.: Sweat gland tumours)

As the skin of the external auditory canal shows a high concentration of ceruminal glands it is susceptible for this already very rare type of benign and malignant tumours. Benign **adnexal tumours **include ceruminous adenomas and pleomorphic adenoma which are best treated by wide local excision [78]. Malignant adnexal tumours include adenoidcystic-, mucinous-, cylindro-, poro-, spiradeno and adenocarcinoma [79-81]. They should be treated by an initial aggressive wide en bloc surgical resection with a primary lateral or subtotal temporal bone resection stage dependent combined with a parotidectomy and neck dissection. Even in T1 tumours local resection is described to be not sufficient [82]. However due to their rarity, a further discussion of these individual tumors is beyond the scope of this article.

##### Blue Nevus (Syn.: Naevus bleu, Tieche's Nevus)

**Figure 13:**
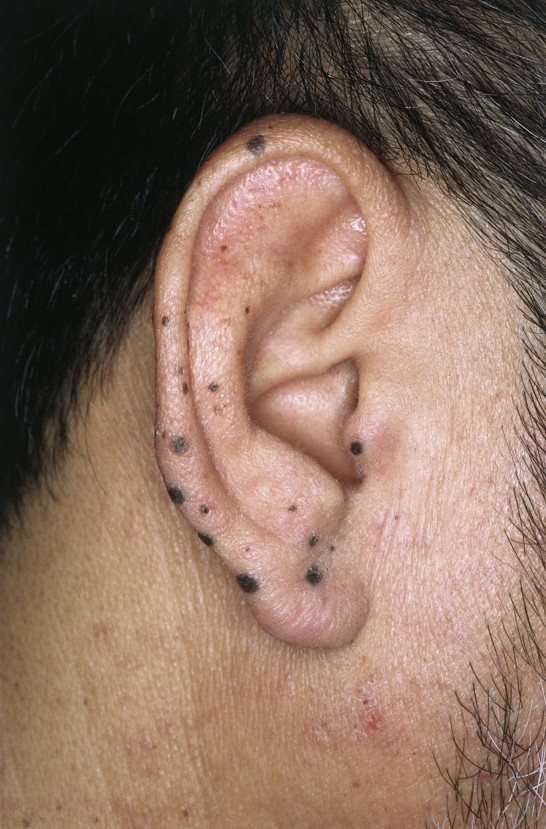
Multiple blue naevus: Multiple, gray to blue, pinhead-sized macules affecting the external ear. If multiple blue naevi appear at the head and neck, remind that rare syndromes (e.g. Carney's syndrome) may be causal.

The **blue nevus **is a variant of a common benign mole which can be clinically misdiagnosed as a melanoma. The lesion is a gray blue to dark blue, smooth surfaced macule which is composed of melanocytes. When multiple blue nevi are located at the head and neck Carney's syndrome, a rare association of blue nevi with hypercortisolism and a variety of nonendocrine (myxomas) and endocrine tumors should be considered (Fig. 13). Solitary, stable lesions can be observed. A changing pigmented lesion should be biopsied to rule out the differential diagnosis of a malignant melanoma.

**Figure 14:**
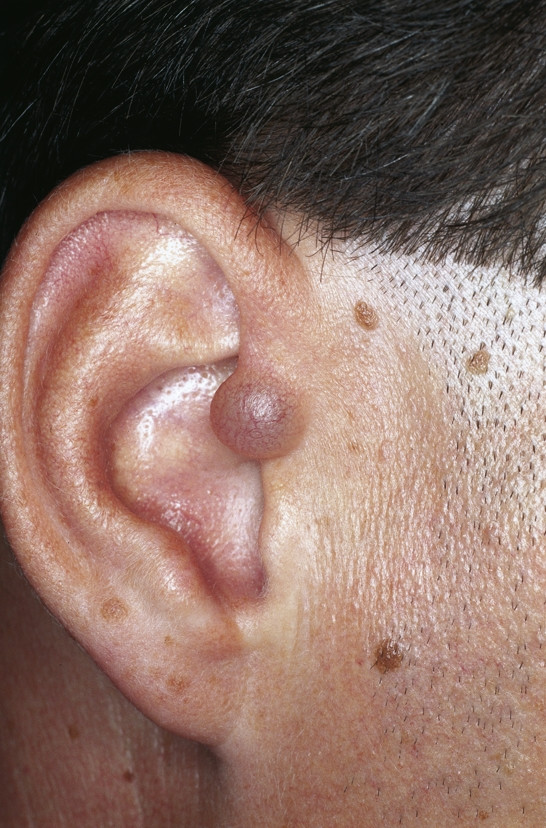
Auricular appandage: Dome-shaped, flesh-colored nodule with smooth surface at the upper part of the tragus.

**Auricular appendages **are themselves harmless lesions (Fig. 14). In very rare cases they can be cutaneous manifestation of a complex disease. Goldenhar syndrome, Wildervanck syndrome, and VACTERL association are very rare diseases which should be considered further. Surgical therapy is only done according to a specific wish of the patient.

**Figure 15:**
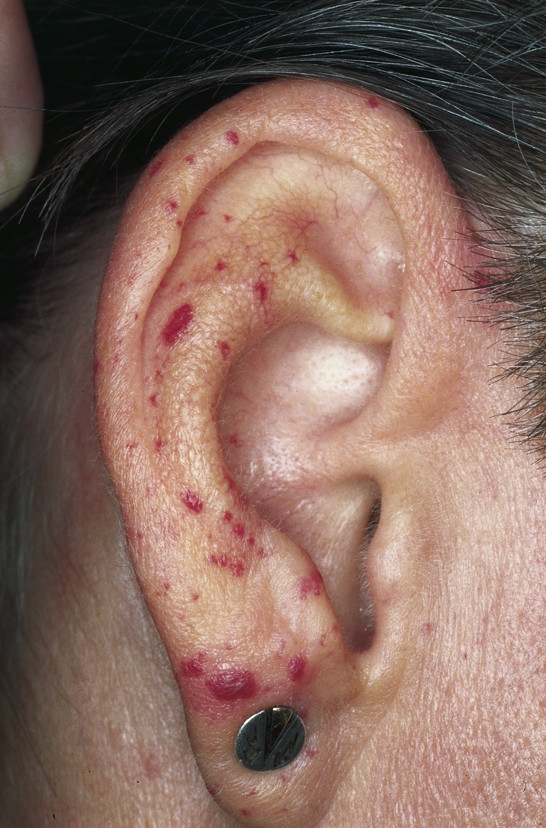
Osler-Weber-Rendu Disease. Multiple, punctuate, red macules and papules corresponding to hemangiomas and telangiectases.

**Osler-Weber-Rendu Disease (Hereditary hemorrhagic teleangiectasia) **is an autosomal dominant disease which manifests itself with multiple punctuate hemangiomas and telangiectases (Fig. 15). Additional characteristics are epistaxis and mucocutaneous visceral arteriovenous malformations. Laser therapy with a long-pulsed Nd:YAG laser, flash-pumped dye laser, or an intense pulsed light (IPL) system has been described as effective.

## Conclusion

The outer ear with the auricle and ear canal can be affected by a variety of different skin lesions and dermatologic conditions. They can be either solitary lesions which are locally limited to the ear or are part of a generalized dermatologic condition. They can afflict skin, cartilage, glands, vessels and hair follicles of the outer ear.

The outer ear itself plays a functional role in audition by collecting and transmitting sound. Additionally it has an important effect on facial appearance and therefore on the individual psychological disposition. Although the auricles skin macroscopically shares the anatomy and physiology of the bodys skin it shows some histological differences compared to the rest of the bodys skin. This specific anatomical peculiarities should be considered when treating skin lesions on the ear.

In cloncusion, the authors suggest that an interdisciplinary approach that combines surgery, dermatology and otolaryngology can provide optimal care for the patient. The most common skin diseases of the outer ear which are potentially subject of surgical or laser therapy have been described briefly in this review.

## Competing interests

The author(s) declare that they have no competing interests.

## Authors' contributions

MS: Documented and prepared the draft. DS: Edited the manuscript, revision of bibliography and helped in preparing the draft. DB: Revised and edited the manuscript. PA: Literature search, and helped with editing of the manuscript. BM: Revised the manuscript, literature search. FGB: Helped in preparing the draft and edited most of the manuscript. All authors read and approved the final manuscript.
